# Asymmetric Cholinergic Basal Forebrain Atrophy Marks Freezing of Gait in Parkinson's Disease

**DOI:** 10.1111/ejn.70467

**Published:** 2026-03-22

**Authors:** Chesney E. Craig, Prabesh Kanel, Nicola J. Ray, Nicolaas I. Bohnen

**Affiliations:** ^1^ Department of Psychology Manchester Metropolitan University Manchester UK; ^2^ Radiology University of Michigan Ann Arbor Michigan USA; ^3^ Morris K. Udall Center of Excellence for Parkinson's Disease Research University of Michigan Ann Arbor Michigan USA; ^4^ Parkinson's Foundation Center of Excellence University of Michigan Ann Arbor Michigan USA; ^5^ Neurology University of Michigan Ann Arbor Michigan USA; ^6^ Neurology Service and GRECC Veterans Administration Ann Arbor Healthcare System Ann Arbor Michigan USA

**Keywords:** cholinergic, freezing of gait, magnetic resonance imaging, Parkinson's disease

## Abstract

Freezing of gait (FoG) and falls are among the most disabling symptoms in late‐stage Parkinson's disease (PD). While right‐lateralised thalamic cholinergic denervation has been linked to FoG and gait impairment, it is unclear whether similar asymmetry exists within the cortically‐projecting cholinergic basal forebrain (cBF), particularly the nucleus basalis of Meynert (Ch4).

In this cross‐sectional study, we assessed structural MRI in 136 nondemented people with PD, stratified into three groups: FoG (*n* = 18), fallers without FoG (*n* = 31) and nonfallers without FoG (*n* = 87). Subregional cBF volumes were quantified using volumetry, normalised by total intracranial volume and compared across groups.

Mixed ANOVAs revealed significantly reduced Ch4 and Ch4p volumes in the FoG group compared to both other groups, with right‐lateralised Ch4p atrophy observed specifically in FoG. After adjusting for disease severity, sex, levodopa equivalent dose (LEDD), and most affected side, the FoG group continued to show significantly reduced volumes in only the right Ch4p. A mediation model indicated that global cognitive performance (MoCA) did not significantly mediate the association between Ch4p volume and FoG status, suggesting that Ch4p degeneration may contribute to FoG through mechanisms not captured by global cognition alone.

Overall, the findings support a right‐hemisphere cholinergic vulnerability in FoG, implicating Ch4p degeneration in networks relevant to both gait regulation and attentional–visuospatial function. Future longitudinal studies are needed to determine whether this lateralised structural vulnerability predicts progression to FoG or cognitive decline in PD.

## Graphical abstract

Participants with freezing of gait (FoG) showed selectively reduced cholinergic basal forebrain volume in the right Ch4p subregion, even after accounting for disease severity, sex, levodopa equivalent dose and most affected side. This association between Ch4p volume and FoG status was not mediated by global cognitive performance (MoCA score). These findings support a right‐hemisphere cholinergic vulnerability in FoG and suggest that longitudinal studies should assess Ch4p degeneration as an early prognostic marker of future FoG.

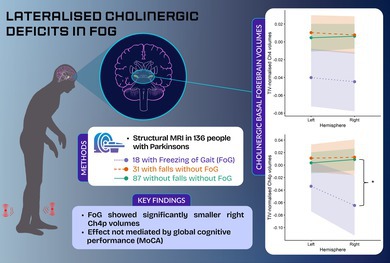

**Abbreviations**
cBF: cholinergic basal forebrain
CSF: cerebrospinal fluid
DAN: dorsal attention network
FoG: freezing of gait
GM: grey matter
LDTC: laterodorsal tegmental complex
LEDD: levodopa equivalent dose
LGN: lateral geniculate nucleus
MoCA: Montreal Cognitive Assessment
MNI: Montreal Neurological Institute
MRI: magnetic resonance imaging
NbM: nucleus basalis of Meynert
PD: Parkinson's Disease
PIGD: postural instability and gait disturbances
PPN: pedunculopontine nucleus
ROI: region(s) of interest
SD: standard deviation
TIV: total intracranial volume
UPDRS III: Unified Parkinson's Disease Rating Scale Part 3
VAN: ventral attention network
WM: white matter

## 1 Introduction

Freezing of gait (FoG) and falls are among the most disabling symptoms in advanced Parkinson's disease (PD) (Port et al. 2021), often marking a more pernicious disease trajectory, including cognitive impairment and early mortality (Bäckström et al. 2018). Both FoG and dopamine‐resistant postural instability and gait disturbances (PIGD) become highly prevalent as PD progresses, affecting up to 80% of patients (Hely et al. 2005; Macht et al. 2007).

Given their limited responsiveness to dopaminergic therapy (Espay et al. 2012), nondopaminergic systems, particularly cholinergic, have been implicated in the pathophysiology of FoG and PIGD (Bohnen et al. 2019; Craig et al. 2020; Gan et al. 2023; Wilson et al. 2021). Importantly, cholinergic pathways are not only critical for motor control but also for cognitive processes such as attention and executive function, which themselves influence gait regulation (Morris et al. 2019a). Thus, cholinergic pathways may represent a shared neural substrate underpinning gait disturbances and cognitive decline in PD.

The brain's cholinergic innervation arises from two major sources: (1) the cholinergic basal forebrain (cBF; Ch1–4) and (2) the brainstem (Ch5 and 6). The cBF provides widespread cortical projections involved in attention, memory and learning (Mesulam, Mufson, et al. 1983). More specifically, Ch1–2 neurons project to the hippocampus, Ch3 neurons project to the olfactory bulb and Ch4 (also known as the nucleus basalis of Meynert [NbM]) neurons project to the cerebral cortex and amygdala (Mesulam, Mufson, et al. 1983), via two distinct and topographically organised tracts, known as the medial and lateral cholinergic pathways (Selden et al. 1998). In contrast, brainstem nuclei (Ch5, pedunculopontine nucleus [PPN], and Ch6, laterodorsal tegmental complex [LDTC]) project to the thalamus, frontal cortex, brainstem, spinal cord and cerebellum (Mesulam, Mufson, et al. 1983).

Both pathways have been implicated in PIGD and/or FoG (Bohnen et al. 2019; Craig et al. 2020; Gan et al. 2023; Wilson et al. 2021), with research suggesting that cholinergic deficits may occur within a broader attentional‐motor network, associated with a failure of attentional and sensorimotor integration (Mesulam, Mufson, et al. 1983). Positron emission tomography (PET) studies show that falls and slow gait speed in PD are associated with PPN/LTDC‐thalamic and cBF‐corticopetal projection denervation (Bohnen et al. 2009, 2013). Notably, falls and FoG may show distinct cholinergic system changes, whereby FoG is associated with diffuse bilateral cholinergic dysfunction, particularly within the striatum, temporal and mesofrontal limbic regions. Meanwhile, falls are linked to right‐lateralised deficits in the visual thalamus, principally within the lateral geniculate nucleus (LGN) (Bohnen et al. 2019).

Structural magnetic resonance imaging (MRI) further implicate degeneration of the PPN (Craig et al. 2020) and/or the cBF, particularly Ch4 (Gan et al. 2023; Wilson et al. 2021), in predicting PIGD and FoG severity. Notably, the posterior subregion of Ch4 (Ch4p) has been shown to longitudinally predict key markers of gait decline, specifically step length and step‐time variability (Wilson et al. 2021). Further evidence suggests that Ch4p may be the earliest cBF nucleus to degenerate in individuals with PD who subsequently develop cognitive impairment; Ch4p predicts global cognitive decline (MoCA) at 2 years, whereas the broader Ch4 region becomes the primary predictor at 5 years (Ray et al. 2018). This pattern is consistent with the posterior‐to‐anterior trajectory of cBF degeneration observed in Alzheimer's disease (Kilimann et al. 2014) and reinforces the potential of cholinergic dysfunction, particularly within Ch4p, as an early prognostic marker for both cognitive and gait deterioration.

Analogous to the PET evidence mentioned above, structural connectivity studies suggest that dysfunctional right hemisphere PPN connectivity is associated with gait deficits (Joza et al. 2022) and/or FoG (Fling et al. 2013) in PD. Similarly, functional connectivity research shows reduced connectivity in right hemisphere executive attention and visual networks (Tessitore et al. 2012). A recent intervention study suggests that improved left and right PPN connectivity may be a marker for gait rehabilitation (Cai et al. 2022), reinforcing the hypothesis that right‐hemisphere cholinergic integrity, closely tied to visuospatial processing (Tessitore et al. 2012), is critical for adaptive gait.

Despite both cBF and PPN being implicated in PD, we do not understand how these nuclei participate in FoG and falls risk together. However, if the right‐lateralised changes seen in the PPN also extend to the cBF, this may represent a broader hemispheric vulnerability underlying FoG and falls.

This study aimed to investigate whether right‐lateralised atrophy of the cBF, particularly Ch4, is associated with FoG and falls in PD. We hypothesised that individuals with FoG and/or falls would show greater right‐hemisphere degeneration of Ch4, consistent with a lateralised cholinergic vulnerability associated with FoG and falls.

## 2 Materials and Methods

### 2.1 Participants

A total of 138 individuals with PD were initially recruited. Of these, 136 participants were included in the present analysis based on adequate image quality for T1‐weighted MRI scans, as well as clearly defined fall and FoG status. All participants met the UK Parkinson's Disease Society Brain Bank clinical diagnostic criteria (Hughes et al. 1992), and those with evidence of large vessel stroke or other intracranial lesions on anatomical imaging were excluded. In addition, participants with missing data were excluded.

#### 2.1.1 Clinical Measures

Motor symptom severity was assessed using the Unified Parkinson's Disease Rating Scale (UPDRS) Part III, administered in the dopaminergic medication ‘OFF’ state (M score = 36.60 ± 13.60). The Montreal Cognitive Assessment (MoCA) was utilised as a measure of cognitive function (M score = 26.12 ± 3.36). The mean disease duration was 6.04 ± 4.86 years.

Fall status was determined as any self‐reported falls in the previous 12 months. FoG status was assessed via clinical examination of item 3.11 (‘Freezing of Gait’) from the MDS‐UPDRS Part III, which provides greater reliability than retrospective self‐report (Snijders et al. 2012).

Eighteen participants were classified as having FoG; of these, 12 also reported falls, while six did not. Because of the small number of participants with FoG but no falls, these subgroups were combined for analysis. Among the 118 participants without FoG, 87 were classified as nonfallers and 31 as fallers. The clinical characteristics for each group can be found in Table 1.

**TABLE 1:** Mean (±SD) values of demographic and clinical characteristics for each FoG/falls status group.

	Nonfallers without FoG (*n* = 87)	Fallers without FoG (*n* = 31)	FoG (*n* = 18)
Sex	65 males, 22 females	24 males, 7 females	16 males, 2 females
Age	66.83 ± 7.33*	65.52 ± 6.45*	72.94 ± 8.74
Disease duration	5.16 ± 4.32**	6.77 ± 5.74	8.94 ± 4.48
UPDRS III	33.90 ± 12.41**	36.31 ± 13.52**	49.92 ± 13.60
Hoehn and Yahr	2.29 ± 0.50**	2.44 ± 0.53*	3.11 ± 0.63
MoCA	27.00 ± 2.60	25.10 ± 3.43***	23.53 ± 4.72****
LEDD (mg/day)	522.60 ± 367.95	751.56 ± 426.78***	702.17 ± 373.34

*Note:* Descriptive checks assessed group differences in sample characteristics. *t*‐tests were employed for parametric descriptive variables, namely, age and UPDRS III. Mann–Whitney *U* was used for all other clinical variables, namely, disease duration, Hoehn and Yahr, MoCA and LEDD.

Abbreviations: LEDD = levodopa equivalent dose, MoCA = Montreal Cognitive Assessment, SD = standard deviation, UPDRS III = Unified Parkinson's Disease Rating Scale Part 3.

* *p* < 0.05 difference to FoG.

** *p* < 0.001 difference to FoG.

*** *p* < 0.05 difference to nonfallers.

**** *p* < 0.001 difference to nonfallers.

### 2.2 Imaging Methods

The volumetric MRI acquisition has been previously described by the authors (Bohnen et al. 2019).

#### 2.2.1 T1‐Weighted Volumetric Image Preprocessing

T1 scans were automatically segmented into grey matter (GM), white matter (WM) and cerebrospinal fluid (CSF) partitions of 1.5‐mm isotropic voxel size using the segmentation routine of the CAT12 toolbox (https://neuro‐jena.github.io/cat/) running under SPM12 (http://www.fil.ion.ucl.ac.uk/spm/software/spm12/). The resulting GM and WM partitions of each subject in native space were then high‐dimensionally registered to Montreal Neurological Institute (MNI) space using DARTEL. The GM segments were then warped using the individual flow fields resulting from the DARTEL registration, and voxel values were modulated for volumetric changes introduced by the high‐dimensional normalisation. Thus, the total amount of GM volume present before warping was preserved. All preprocessed GM maps were required to pass a visual inspection for overall segmentation and registration accuracy. This resulted in removal of two participants' images. Voxel values from subject‐specific MNI space GM maps within each of the cBF regions of interest (ROIs) were extracted. In order to ensure all voxels in the final mask represented GM, any values below 0.2 from these maps were rejected. Mean volume was calculated as all remaining voxel values within each ROI.

#### 2.2.2 Regions of Interest

We used the map of the cBF created in Kilimann et al. (2014). The stereotactic cBF map distinguishes different cholinergic subdivisions, including regions that correspond to the medial septum and vertical limb of the diagonal band (Ch1–2 in Mesulam's nomenclature (Mesulam, Mufson, et al. 1983)), horizontal limb of the diagonal band (Ch3), anterior and intermediate NbM (Ch4a–i), the anterolateral NbM/nucleus subputaminalis (Ch4al/NSP) and posterior parts of the NbM (Ch4p). For the purpose of this paper, we separated the Kilimann mask into the left and right hemispheres and extracted volumes for the whole left/right NbM (Ch4; calculated as the sum of Ch4a‐I, Ch4al and Ch4p) and left/right posterior NbM, Ch4p. In addition, left and right Ch1–2 were selected as a control region due to its primary projections to the hippocampus, which are less directly implicated in motor and visuospatial functions relevant to gait.

**FIGURE 1:**
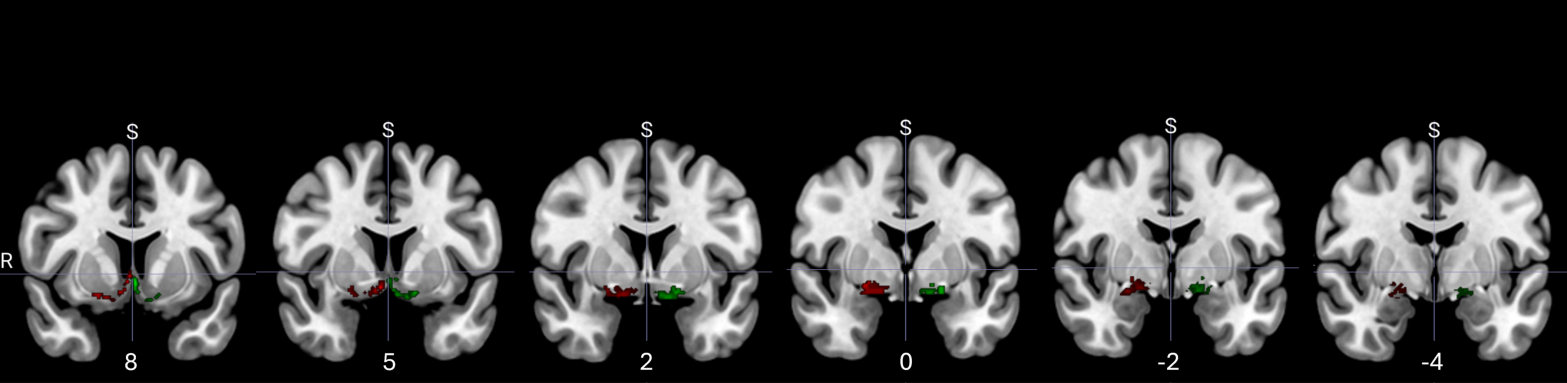
Coronal slices from anterior to posterior showing the left (green) and right (red) segmentation of the Kilimann mask (2014) of the cBF subregions. Numbers below each slice indicate Montreal Neurological Institute (MNI) standard space coordinates (MNI152 template).

The localisation of the mask in MNI space (MNI152 template) can be seen in Figure 1. Mean volumes were calculated for each ROI and normalised via analysis of covariance by total intracranial volume (TIV; calculated as the sum of GM, WM and CSF).

### 2.3 Statistical Analysis

All analyses were conducted in SPSS v27. Group characteristics are reported for descriptive purposes to inform interpretability rather than to test hypotheses. Descriptive checks were performed to explore differences in sample characteristics across groups. For continuous variables with approximately normal distributions (age and UPDRS III), independent‐samples *t*‐tests were used. For nonparametric clinical variables, Mann–Whitney *U* tests were applied. No multiple‐comparison corrections were performed, as these analyses were not hypothesis‐driven.

Mixed ANOVAs, with fall/FoG status as the between‐subject factor and hemisphere as the repeated measures factor, were used to assess differences in cBF volumes for each ROI (Ch4, Ch4p and Ch1–2). Where significant effects were observed, follow‐up analyses included mixed ANCOVAs and simple effects tests, adjusting for relevant covariates: disease severity (adjusted UPDRS III), sex, LEDD and most affected side. To extract a measure of disease severity that was not related to the symptoms associated with FoG itself, an adjusted UPDRS III score was calculated by subtracting the PIGD subscore from the total UPDRS III score.

Given evidence that Ch4p may be an early site of degeneration in individuals who later develop cognitive decline and that cognitive impairment is common in FoG, we conducted a mediation analysis using the PROCESS macro for SPSS (Hayes 2022). This analysis tested whether the relationship between Ch4p volumes and FoG status was mediated by global cognitive performance (MoCA score). Consistent with the ANCOVA, disease severity (adjusted UPDRS III), sex, LEDD and most affected side were included as covariates in the mediation model (Model 4).

### 2.4 Ethical Standards

The protocol for this study received prior ethical approval by the institutional review boards of the University of Michigan and Ann Arbor VA Healthcare and was completed in compliance with the World Medical Association Declaration of Helsinki. Written informed consent was obtained from all participants. Previous findings from this dataset have been published by the authors (Bohnen et al. 2019; Ray et al. 2023).

## 3 Results

### 3.1 Clinical Characteristics

Group‐level clinical characteristics are presented in Table 1. Participants with FoG exhibited higher UPDRS Part III scores compared to both fallers without FoG (*p* < 0.001) and nonfallers (*p* < 0.001). They also had higher Hoehn and Yahr stages (*p* = 0.004 and *p* < 0.001, respectively) and were older than both comparison groups (*p* = 0.001 and *p* = 0.002, respectively). Disease duration was longer in the FoG group compared to nonfallers without FoG (*p* < 0.001), but not fallers.

Cognitive performance, as measured by MoCA, was lower in both the FoG group (*p* < 0.001) and fallers without FoG (*p* = 0.006) compared to nonfallers. Additionally, fallers without FoG had higher LEDD than nonfallers (*p* = 0.003).

### 3.2 Impact of FoG/Fall Status on Lateralised cBF Subregional Atrophy

#### 3.2.1 Ch4 Volumes

A mixed ANOVA with FoG/fall status as the between‐subjects factor (FoG, fallers without FoG and nonfallers) and hemisphere as the within‐subjects factor revealed a significant main effect of group on Ch4 volume (*F*_*2, 133*_ = 4.58, *p* = 0.012). Bonferroni post hoc comparisons revealed that participants with FoG (mean = −0.0401 [SD = 0.699]) had significantly smaller Ch4 volumes than both fallers (mean = 0.0103 [SD = 0.536]; *p* = 0.023) and nonfallers (mean = 0.0046 [SD = 0.738]; *p* = 0.014).

**FIGURE 2:**
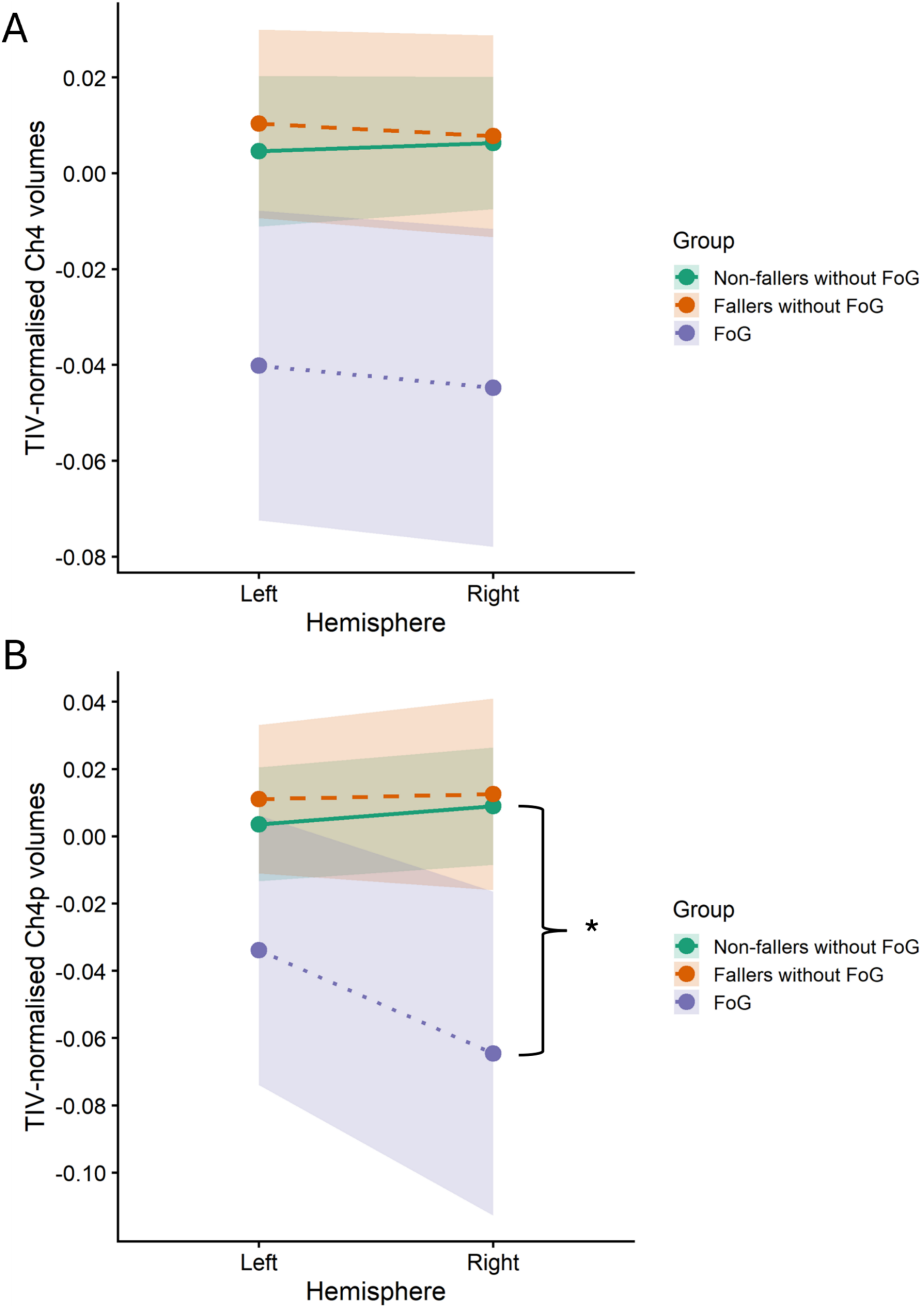
Interaction plots of TIV‐normalised CBF volumes for the (A). Ch4 and (B). Ch4p subregions, for the left and right hemisphere for nonfallers without FoG (*n* = 87), fallers without FoG (*n* = 31) and FoG (*n* = 18). Shaded regions represent 95% confidence intervals.

This group effect was no longer significant when controlling for disease severity, sex, LEDD and most affected side in a mixed ANCOVA (*F*_2, 129_ = 2.69, *p* = 0.071). No significant group by hemisphere interaction was observed (Figure 2A).

#### 3.2.2 Ch4p Volumes

For Ch4p, there was a main effect of group (*F*_*2,133*_ = 4.47, *p* = 0.013), with people with FoG having smaller Ch4p volumes than people with falls (*p* = 0.022, 95% CI = −0.115 to −0.007) and without falls (*p* = 0.016, 95% CI = −0.103 to −0.008). A significant group by hemisphere interaction was also found (*F*_*2,133*_ = 4.41, *p* = 0.014). Simple effects analysis revealed that this effect was specific to the right hemisphere (*F*_*2, 133*_ = 6.33, *p* = 0.002) (Figure 2B).

Bonferroni post hoc comparisons showed that participants with FoG (mean = −0.0646 [SD = 0.0969]) had significantly smaller right Ch4p volumes than both fallers (mean = 0.0126 [SD = 0.0776]; *p* = 0.006, 95% CI = −0.137 to −0.018) and nonfallers (mean = 0.0090 [SD = 0.0817]; *p* = 0.002, 95% CI = −0.126 to −0.022). These differences remained significant after covariate adjustment in a univariate ANCOVA (*F*_*2, 129*_ = 2.976, *p* = 0.054), controlling for disease severity, sex, LEDD and most affected side. Following adjustment, Bonferroni post hoc comparisons indicated that right Ch4p volume was significantly reduced in the FoG group compared with nonfallers only (*p* = 0.044, 95% CI = −0.110 to −0.001). In addition, the FoG group was the only group to show significantly smaller right compared with left Ch4p volumes (*p* = 0.022, 95% CI = −0.003 to −0.032).

#### 3.2.3 Ch1–2 Volumes

No significant main effects or interactions were observed for Ch1–2 volumes.

### 3.3 Testing Cognitive Mediation of the Ch4p–FoG Link

A mediation analysis was conducted using PROCESS (Model 4) with 5000 bootstrap samples. The indirect effect of Ch4p volume on FoG status through global cognitive function (MoCA) was not significant, *ab* = −1.547, 95% bootstrap CI [−8.091, 1.048], as the confidence interval included zero. However, the direct effect of Ch4p volume on FoG status, controlling for MoCA, was significant, *c'* = −11.856, *p* = 0.028. These findings suggest that the association between Ch4p volume and FoG status is not mediated by global cognitive function Table 2.

**TABLE 2:** Mediation analysis examining the indirect effect of Ch4p volume on freezing of gait (FoG) status via global cognition (MoCA).

Effect/path	Predictor → outcome	B	SE	95% bootstrap CI	p
**Path a**	Ch4p volume → MoCA	5.647	3.348	[−0.977, 12.272]	0.094
**Path b**	MoCA → FoG (controlling for Ch4p)	−0.274	0.108	[−0.485, −0.063]	0.011*
**Direct effect (c')**	Ch4p volume → FoG (controlling for MoCA)	−11.856	5.386	[−22.414, −1.303]	0.028*
**Indirect effect (ab)**	Ch4p → MoCA → FoG	−1.547	46.424	[−8.091, 1.048]	—

*Note:* All paths control for disease severity (adjusted UPDRS III), sex, LEDD and most affected side. Unstandardised regression coefficients (*B*) are reported. Standard error (SE) and confidence intervals (CIs) for the indirect effect are bias‐corrected bootstrap outcomes based on 5000 resamples. The indirect effect is considered statistically significant when the 95% confidence interval does not include zero.

Abbreviations: FoG = freezing of gait, MoCA = Montreal Cognitive Assessment.

* *p* < 0.05 and 95% confidence interval does not include zero.

## 4 Discussion

This study examined regional cholinergic basal forebrain (cBF) atrophy in PD in relation to FoG and fall status. The most robust finding was evidence for right‐lateralised atrophy of the posterior nucleus basalis of Meynert (Ch4p) in people with FoG. This region is known to degenerate first in neurodegenerative conditions like Alzheimer's disease (Kilimann et al. 2014). Although initial models indicated reduced total Ch4 and Ch4p volumes in participants with FoG relative to those without FoG, only the right Ch4p reduction remained statistically significant after adjusting for disease severity, sex, LEDD and most affected side. No group differences were found for Ch1–2 volumes, supporting the regional specificity of the Ch4p finding.

The pattern of right‐hemisphere vulnerability aligns with converging evidence of right hemisphere cholinergic dysregulation in PD‐related gait disturbances and FoG (Bohnen et al. 2019; Cai et al. 2022; Fling et al. 2013; Joza et al. 2022; Roytman et al. 2023). Fling et al. (2013) report distinct cholinergic asymmetries in the PPN network in FoG, revealing reduced connectivity between the PPN and the right hemisphere's locomotor network (cerebellum and thalamus) and prefrontal regions involved in executive function. The current findings extend this literature by demonstrating structural compromise of the right cortical cholinergic projections from Ch4p, which may contribute to attentional and visuospatial dysfunction underlying FoG.

Previous work from the ICICLE‐GAIT cohort identified Ch4p degeneration as a longitudinal predictor of disease‐specific gait decline (Wilson et al. 2021). Until now, however, this subregion had not been directly linked to FoG. Given the strong clinical and neurobiological overlap between FoG and cognitive impairment (Monaghan et al. 2023), Ch4p could be a potential cholinergic substrate underpinning shared neuropathological mechanisms across these domains (Morris et al. 2019). This is particularly relevant because Ch4p integrity has also been suggested as an early prognostic marker for later cognitive decline (Ray et al. 2018).

Despite this, our mediation analysis indicated that global cognitive performance (MoCA) did not significantly mediate the association between Ch4p volume and FoG status. This suggests that Ch4p atrophy contributes to FoG through pathways not captured by global cognition alone. Instead, degeneration in this region may reflect broader cholinergic dysfunction involving attentional or visuospatial circuits, as well as broader corticopetal projections involved in gait‐relevant cortical processes such as sensory integration. Accordingly, the link between Ch4p integrity and FoG appears to be at least partly direct, underscoring the role of cholinergic pathways in FoG pathophysiology.

It is interesting to note that the current paper did not find right lateralising effects in fallers without FoG, despite previous reports of right thalamic cholinergic deficits in this group (Bohnen et al. 2019; Cai et al. 2022; Joza et al. 2022). This could support a right hemisphere vulnerability as Parkinson's progresses and PIGD symptoms and freezing emerge, which is more prevalent in PPN‐thalamic cholinergic projections for fallers without FoG and then extends to cBF‐corticopetal projections for FoG. These projections likely contribute to the executive attention and visual networks shown to be disrupted in FoG (Tessitore et al. 2012).

Recent work from our group (2023) supports right hemisphere dominant cholinergic dysfunction in FoG. The study employed cholinergic PET and computerised posturography, which permitted assessment of postural instability under various conditions of sensory conflict in people with PIGD with and without FoG. The findings suggested that cholinergic loss in the right hemisphere was associated with impaired vestibular multisensory integration. Future work should investigate if FoG is mediated by loss of vestibular function as a result of right hemisphere cholinergic loss.

Research suggests that PIGD symptoms may be associated with a more general vulnerability of right hemisphere circuitry (Peterson et al. 2014; Snijders et al. 2012). Previous authors (Fling et al. 2013) have argued that this lateralisation may be linked to the right hemisphere's specialisation in spatial cognition, body schema and action inhibition, all of which are functionally relevant to adaptive gait modulation.

A recent post‐mortem stereological analysis of cholinergic neurons with the bilateral PPN found evidence of more severe cholinergic neuronal loss in right‐sided PPNs, potentially driving symptom lateralisation (Sharma et al. 2025). A right hemisphere vulnerability would also implicate the ventral attention network (VAN), which is lateralised to the right, comprising the temporoparietal junction, inferior frontal gyrus, anterior insula, frontal operculum and anterior cingulate cortex (Corbetta and Shulman 2011). This network is thought to modulate bottom‐up involuntary attention to salient stimuli and disruption to this network is associated with spatial neglect (Corbetta and Shulman 2011). Clinical evidence (Ebersbach et al. 1996) suggests a subtle left hemineglect in Parkinson's, whilst more recent research suggests disruption to the VAN and associated involuntary attention deficits as the disease progresses (Martínez‐Serrato et al. 2023). On the other hand, Maidan et al. (2019) found that the dorsal attention network (DAN), rather than the VAN, was associated with FoG. Our findings of right‐sided Ch4p atrophy in FoG could support contributions from both VAN and DAN, given their overlap with the medial and lateral cholinergic branches. Further research is needed to investigate possible asymmetric cholinergic neuronal losses in Ch4(p) and to delineate their contributions to FoG. Post‐mortem stereological analyses are biased by end‐stage disease and as suggested by our clinical and demographic data, FoG typically represents a more advanced cholinergic degeneration state than PD fallers without FoG.

### 4.1 Limitations

Several limitations should be acknowledged. First, FoG assessment did not incorporate a dedicated provocative protocol to capture specific freezing subtypes, which may account for the relatively low prevalence of FoG in our sample. As such, the reported effects are likely conservative, and future studies should employ more sensitive FoG assessments, such as 360° turning tasks (Zoetewei et al. 2024). Similarly, fall status was based on retrospective self‐report over 12 months, a method known to underestimate falls compared with prospective monitoring (Freiberger and de Vree 2011). Larger studies using both provocative FoG testing and prospective fall recording are needed to confirm the present findings.

Our analyses focused on the whole NbM (combined Ch4a‐I, Ch4al and Ch4p) and the Ch4p subdivision, given their prior associations with FoG and PIGD. Future work, using scanning procedures optimised for higher‐resolution imaging of the cholinergic basal forebrain, could extend this by examining each cBF subdivision separately. Finally, we cannot exclude the influence of other neuropsychiatric comorbidities, such as anxiety or depression, which may differentially affect individuals with FoG. Including these variables in future models will help clarify their potential confounding effects.

## 5 Conclusions

In summary, this is the first study to demonstrate that right‐lateralised degeneration of the cholinergic basal forebrain, particularly within the Ch4p subregion, is associated with FoG in Parkinson's. Although individuals with FoG showed broader reductions in Ch4 and Ch4p volumes, only right‐hemisphere Ch4p atrophy remained significant after accounting for clinical covariates, highlighting its regional specificity. Importantly, global cognitive function did not mediate the relationship between Ch4p volume and FoG, suggesting that Ch4p degeneration contributes to FoG through mechanisms not captured by global cognitive screening. Instead, these findings point to disruption of right‐hemisphere cholinergic pathways supporting attentional, visuospatial and sensory‐integration processes essential for gait control.

Future longitudinal studies should determine whether asymmetric cholinergic degeneration predicts the onset or worsening of FoG and whether it also contributes to later cognitive decline. Clarifying these pathways may help inform future research into targeted therapeutic strategies aimed at preserving right hemisphere cholinergic function to mitigate gait dysfunction in PD.

## Author Contributions

**Chesney E. Craig:** formal analysis, visualization, writing – original draft, writing – review and editing. **Prabesh Kanel:** data curation, investigation, methodology, writing – review and editing. **Nicola J. Ray:** conceptualization, formal analysis, supervision, visualization, writing – review and editing. **Nicolaas I. Bohnen:** conceptualization, funding acquisition, methodology, project administration, supervision, visualization, writing – review and editing.

## Funding

This work was supported by the National Institutes of Health (P01 NS015655, P50 NS091856, P50 NS123067, R01 AG073100 and RO1 NS070856), Michael J. Fox Foundation for Parkinson's Research, Farmer Family Foundation. Parkinson's Foundation and the Department of Veterans Affairs (I01 RX003397).

## Conflicts of Interest

The authors declare conflicts of interest.

## Data Availability

The data that support the findings of this study are available from the corresponding author, upon reasonable request.
